# Supplementary material for: Genome-Wide Association Studies (GWAS) Approaches for the Detection of Genetic Variants Associated with Antibiotic Resistance: A Systematic Review
Source: Microorganisms. 2023 Nov 27;11(12):2866. doi: 10.3390/microorganisms11122866 (PMC10745584; doi:10.3390/microorganisms11122866)
Supplement: Supplementary file 1 [file microorganisms-11-02866-s001.zip › Supplementary Material File S1 PRISMA_2020_checklist.pdf]

PRISMA 2020

PRISMA 2020 Checklist

Section and Topic	Item #	Checklist item	Location where item is reported
**TITLE**			
Title	1	Identify the report as a systematic review.	Page 1. **"Genome-wide association studies (GWAS) approaches for the detection of genetic variants associated with antibiotic resistance: a systematic review".**
**ABSTRACT**			
Abstract	2	See the PRISMA 2020 for Abstracts checklist.	Page 1.
**INTRODUCTION**			
Rationale	3	Describe the rationale for the review in the context of existing knowledge.	Page 1."**The primary purpose of GWAS is to identify statistically significant associations that may indicate causal relationships between genotype and phenotype while eliminating spurious associations arising from confounding factors (Collins et al., 2018). Consequently, there is an important interest in implementing GWAS approaches in pathogenic bacteria to advance our understanding of infectious disease risks and identify genetic variants driving bacterial resistance (Power et al., 2016; Chen, 2015; Read et al., 2014; Jaillard et al., 2018), offering immense potential for substantial improvements in disease management and treatment"**.
Objectives	4	Provide an explicit statement of the objective(s) or question(s) the review addresses.	Page 2. **"The objective of this systematic review is to describe bacterial GWAS approaches employed to identify genetic variants associated with antimicrobial resistance, as well as alternative strategies for discerning genotype-phenotype associations in bacteria".**
**METHODS**			
Eligibility criteria	5	Specify the inclusion and exclusion criteria for the review and how studies were grouped for the syntheses.	Page 2. **"The eligibility criteria for inclusion in the review will be based on the PICOS (Population, Intervention, Comparison, Outcome, and Study design) framework: 1) Population will be bacterial species that are known to cause infections in humans or an-imals. 2) Interventions will be genome-wide association studies to identify bacterial resistance determinants. 3) Comparison groups will be non-GWAS studies that have in-vestigated bacterial resistance determinants. 4) Outcome will be the genetic variants associated**

Section and Topic	Item #	Checklist item	Location where item is reported
			**with bacterial resistance. 5) Study design will be genome-wide association studies published in peer-reviewed journals. Additionally, only publications in English will be considered. Studies reporting on phenotypes other than antibiotic resistance will be excluded. Conference abstracts, editorials, review articles, book chapters, and studies that applied GWAS approaches in organisms other than bacteria (viruses, fungi, humans) will also be excluded”**.
Information sources	6	Specify all databases, registers, websites, organisations, reference lists and other sources searched or consulted to identify studies. Specify the date when each source was last searched or consulted.	Page 2, **S**upplementary Material Table S2. **“The search will be conducted on major databases, such as PubMed and Scopus, which are widely used repositories of scientific literature. To ensure comprehensiveness, a mix of MeSH terms and keywords will be employed, including "genome-wide association study", "antibiotic resistance", “antimicrobial resistance” and "GWAS". Supplementary Material Table S2 will provide a list of the search strategies tailored to the specific requirements of each database. Only articles published in the full text before February 7, 2023”**.
Search strategy	7	Present the full search strategies for all databases, registers and websites, including any filters and limits used.	Supplementary Material Table S2. “**Supplementary Material Table S2 will provide a list of the search strategies tailored to the specific requirements of each database. Only articles published in the full text before February 7, 2023”**.
Selection process	8	Specify the methods used to decide whether a study met the inclusion criteria of the review, including how many reviewers screened each record and each report retrieved, whether they worked independently, and if applicable, details of automation tools used in the process.	Page 3. “**A single reviewer will conduct a two-phase review of the studies. The first phase will involve an initial screening of the articles to evaluate the relevance of the title and abstract. During this phase, the reviewer will identify those articles that meet the inclusion criteria. In the second phase, the reviewer will thoroughly evaluate the full text of the articles that passed the initial screening to determine if they meet the eligibility criteria. The same inclusion criteria will be applied during both phases of the selection process to ensure consistency in the review and minimize bias. The reviewer will also document the reasons for excluding articles that do not meet the inclusion criteria**”.
Data collection process	9	Specify the methods used to collect data from reports, including how many reviewers collected data from each report, whether they worked independently, any processes for obtaining or confirming data from study investigators, and if applicable, details of automation tools used in the process.	Page 3. **“An Excel spreadsheet will be utilized to gather information from each GWAS study in the systematic review”. “All citations and**

Section and Topic	Item #	Checklist item	Location where item is reported
			**publications abstracts obtained from the results of the search equations in Scopus and PubMed will be exported to Rayyan (https://rayyan.qcri.org), a web application designed for the systematic review of articles. Using this tool, the entire selection process, including the review of titles and abstracts, elimination of duplicate articles, and selection of studies that meet the eligibility criteria, will be managed".**
Data items	10a	List and define all outcomes for which data were sought. Specify whether all results that were compatible with each outcome domain in each study were sought (e.g. for all measures, time points, analyses), and if not, the methods used to decide which results to collect.	Page 3. **"The essential data to be collected will comprise study characteristics, authors, year of study, the objective of the study, bacterial species studied, sample size, resistance phenotype, phenotypic traits, genetic variants associated with antibiotic resistance, GWAS software used, GWAS approach employed, population structure control methods (useful for analyzing the statistical methods employed in the studies), parameter statistics (multiple test corrections), significant genetic variants identified, findings relevant, validation of the results found, and any additional relevant data that can provide a comprehensive understanding of the studies".**
	10b	List and define all other variables for which data were sought (e.g. participant and intervention characteristics, funding sources). Describe any assumptions made about any missing or unclear information.	Page 3. **"The essential data to be collected will comprise study characteristics, authors, year of study, the objective of the study, bacterial species studied, sample size, resistance phenotype, phenotypic traits, genetic variants associated with antibiotic resistance, GWAS software used, GWAS approach employed, population structure control methods (useful for analyzing the statistical methods employed in the studies), parameter statistics (multiple test corrections), significant genetic variants identified, findings relevant, validation of the results found, and any additional relevant data that can provide a comprehensive understanding of the studies".**
Study risk of bias assessment	11	Specify the methods used to assess risk of bias in the included studies, including details of the tool(s) used, how many reviewers assessed each study and whether they worked independently, and if applicable, details of automation tools used in the process.	NA
Effect measures	12	Specify for each outcome the effect measure(s) (e.g. risk ratio, mean difference) used in the synthesis or presentation of results.	NA
Synthesis	13a	Describe the processes used to decide which studies were eligible for each synthesis (e.g. tabulating the study intervention characteristics and comparing against the planned groups for each synthesis	NA

Section and Topic	Item #	Checklist item	Location where item is reported
methods		(item #5)).	
	13b	Describe any methods required to prepare the data for presentation or synthesis, such as handling of missing summary statistics, or data conversions.	NA
	13c	Describe any methods used to tabulate or visually display results of individual studies and syntheses.	NA
	13d	Describe any methods used to synthesize results and provide a rationale for the choice(s). If meta-analysis was performed, describe the model(s), method(s) to identify the presence and extent of statistical heterogeneity, and software package(s) used.	NA
	13e	Describe any methods used to explore possible causes of heterogeneity among study results (e.g. subgroup analysis, meta-regression).	NA
	13f	Describe any sensitivity analyses conducted to assess robustness of the synthesized results.	NA
Reporting bias assessment	14	Describe any methods used to assess risk of bias due to missing results in a synthesis (arising from reporting biases).	NA
Certainty assessment	15	Describe any methods used to assess certainty (or confidence) in the body of evidence for an outcome.	NA
**RESULTS**			
Study selection	16a	Describe the results of the search and selection process, from the number of records identified in the search to the number of studies included in the review, ideally using a flow diagram.	Figure 1
	16b	Cite studies that might appear to meet the inclusion criteria, but which were excluded, and explain why they were excluded.	Page 4. **“Furthermore, studies that conducted GWAS in non-bacterial species (186 studies) or in the incorrect pheno-type (50 studies) were also excluded. In addition, studies that were not of the correct publication type were excluded, which included review articles, case reports, and conference abstracts (37 studies). After the initial screening process, 57 articles were left for full-text evaluation. During the full-text evaluation, articles that did not meet the inclusion criteria were excluded, resulting in the removal of 17 studies. Out of the 17 excluded studies, 8 did not use GWAS, and 9 used a phenotype other than antibiotic resistance”**.
Study characteristics	17	Cite each included study and present its characteristics.	Table 2
Risk of bias in studies	18	Present assessments of risk of bias for each included study.	NA
Results of individual studies	19	For all outcomes, present, for each study: (a) summary statistics for each group (where appropriate) and (b) an effect estimate and its precision (e.g. confidence/credible interval), ideally using structured tables or plots.	NA
Results of syntheses	20a	For each synthesis, briefly summarise the characteristics and risk of bias among contributing studies.	NA
	20b	Present results of all statistical syntheses conducted. If meta-analysis was done, present for each the summary estimate and its precision (e.g. confidence/credible interval) and measures of statistical	NA

Section and Topic	Item #	Checklist item	Location where item is reported
		heterogeneity. If comparing groups, describe the direction of the effect.	
	20c	Present results of all investigations of possible causes of heterogeneity among study results.	NA
	20d	Present results of all sensitivity analyses conducted to assess the robustness of the synthesized results.	NA
Reporting biases	21	Present assessments of risk of bias due to missing results (arising from reporting biases) for each synthesis assessed.	NA
Certainty of evidence	22	Present assessments of certainty (or confidence) in the body of evidence for each outcome assessed.	NA
**DISCUSSION**			
Discussion	23a	Provide a general interpretation of the results in the context of other evidence.	Page 17-19. F**or example: "The studies collected in this review reveal a wide variety of GWAS approaches including non-phylogenetic, phylogenetic, alignment-free *k*-mers-based, and mixed approaches that significantly contribute to the comprehension of genetic components associated with bacterial phenotypic traits, particularly concerning antibiotic resistance. These findings highlight the evolution that characterizes this field of research, distinguished by significant advances and concurrent challenges. As we deepen our com-prehension of genotype-phenotype correlations, novel methodologies surface to sur-mount the constraints of conventional GWAS methodologies, thereby facilitating a more comprehensive understanding of bacterial resistance mechanisms".**
	23b	Discuss any limitations of the evidence included in the review.	
	23c	Discuss any limitations of the review processes used.	Page 19. "**Our review was not without some limitations. To focus on the GWAS approaches used to detect genetic variants associated with antibiotic resistance, we excluded those studies that performed GWAS with a different bacterial phenotype. It is possible that studies that used different GWAS approaches that could be applied in the analysis of the antimicrobial-resistant phenotype have not been considered".**
	23d	Discuss implications of the results for practice, policy, and future research.	**Page 19. "In summary, despite that diverse GWAS approaches have been applied in re-search aiming to identify genetic variants associated with antimicrobial resistance to understand different bacterial mechanisms for developing resistance to available drugs reported in this review. There is a need to continue working on**

Section and Topic	Item #	Checklist item	Location where item is reported
			**challenges, including epistasis, compensatory mutations, gene-gene interactions, and environmental factors that may influence the understanding of the mechanisms that contribute to the devel-opment of drug resistance. Additionally, another area with considerable potential for exploration is the development of novel computational methods that combine GWAS approaches with machine learning techniques. At the same time, it is necessary to in-tegrate omics data, such as metabolomics, metabolic networks, and protein structural data, into a single analysis. Without a doubt, it could accelerate our understanding of the biological mechanisms of response to drugs, which contributes to the way we ad-dress the public health problem which is resistance to available antibiotics.”**
**OTHER INFORMATION**			
Registration and protocol	24a	Provide registration information for the review, including register name and registration number, or state that the review was not registered.	NA
	24b	Indicate where the review protocol can be accessed, or state that a protocol was not prepared.	NA
	24c	Describe and explain any amendments to information provided at registration or in the protocol.	NA
Support	25	Describe sources of financial or non-financial support for the review, and the role of the funders or sponsors in the review.	Page 20.
Competing interests	26	Declare any competing interests of review authors.	Page 20. **“The authors declare no conflict of interests”.**
Availability of data, code and other materials	27	Report which of the following are publicly available and where they can be found: template data collection forms; data extracted from included studies; data used for all analyses; analytic code; any other materials used in the review.	Page 20. **“The data presented in this study are available in the supplementary materials”**.

PRISMA 2020 for Abstracts Checklist

Section and Topic	Item #	Checklist item	Reported (Yes/No)
**TITLE**			
Title	1	Identify the report as a systematic review.	Yes
**BACKGROUND**			
Objectives	2	Provide an explicit statement of the main objective(s) or question(s) the review addresses.	Yes
**METHODS**			
Eligibility criteria	3	Specify the inclusion and exclusion criteria for the review.	No
Information sources	4	Specify the information sources (e.g. databases, registers) used to identify studies and the date when each was last searched.	Yes
Risk of bias	5	Specify the methods used to assess risk of bias in the included studies.	NA
Synthesis of results	6	Specify the methods used to present and synthesise results.	No
**RESULTS**			
Included studies	7	Give the total number of included studies and participants and summarise relevant characteristics of studies.	Yes
Synthesis of results	8	Present results for main outcomes, preferably indicating the number of included studies and participants for each. If meta-analysis was done, report the summary estimate and confidence/credible interval. If comparing groups, indicate the direction of the effect (i.e. which group is favoured).	Yes
**DISCUSSION**			
Limitations of evidence	9	Provide a brief summary of the limitations of the evidence included in the review (e.g. study risk of bias, inconsistency and imprecision).	No
Interpretation	10	Provide a general interpretation of the results and important implications.	Yes
**OTHER**			
Funding	11	Specify the primary source of funding for the review.	No
Registration	12	Provide the register name and registration number.	No

From: Page MJ, McKenzie JE, Bossuyt PM, Boutron I, Hoffmann TC, Mulrow CD, et al. The PRISMA 2020 statement: an updated guideline for reporting systematic reviews. BMJ 2021;372:n71. doi: 10.1136/bmj.n71
