# Supplementary material for: Genome-Wide Association Studies (GWAS) Approaches for the Detection of Genetic Variants Associated with Antibiotic Resistance: A Systematic Review
Source: Microorganisms. 2023 Nov 27;11(12):2866. doi: 10.3390/microorganisms11122866 (PMC10745584; doi:10.3390/microorganisms11122866)
Supplement: Supplementary file 1 [file microorganisms-11-02866-s001.zip › Supplementary Material Table S1 Search Strategies.pdf]

Supplementary Material Table S2: Search Strategies

Database PubMed			
**Search**	**Query**	**Number of Results**	**Date search**
1	(Bacteria OR Bacterial) AND ("genome wide association" OR "whole genome association" OR "GWAS") AND ("drug resistance" OR "antimicrobial resistance" OR "phenotypic resistance" OR "minimum inhibitory concentration" OR quantitative resistance phenotypes OR "Minimal inhibitory concentration")	376	26-08-2022
2	(Bacteria OR Bacterial) AND ("genome wide association" OR "whole genome association" OR "GWAS") AND ("drug resistance" OR "antimicrobial resistance" OR "phenotypic resistance" OR "minimum inhibitory concentration" OR quantitative resistance phenotypes OR "Minimal inhibitory concentration")	391	08-02-2023
**Total (After Duplicates Removed)**		**395**	

Database Scopus			
**Search**	**Query**	**Number of Results**	**Date search**
1	ALL ("Bacteria" OR "Bacterial") AND ALL ("genome wide association" OR "whole genome association" OR "GWAS") AND ALL ("drug and resistance" OR "antimicrobial and resistance" OR "phenotypic and resistance" OR "minimum inhibitory concentration" OR "quantitative resistance phenotypes" OR "Minimal inhibitory concentration")	264	26-08-2022
2	ALL ("Bacteria" OR "Bacterial") AND ALL ("genome wide association" OR "whole genome association" OR "GWAS") AND ALL ("drug and resistance" OR "antimicrobial and resistance" OR "phenotypic and resistance" OR "minimum inhibitory concentration" OR "quantitative resistance phenotypes" OR "Minimal inhibitory concentration")	316	08-02-2023
**Total (After Duplicates Removed)**		**252**	
